# Kidney and combined kidney and pancreas transplantation may be under-utilized in cystic fibrosis

**DOI:** 10.3389/frtra.2022.992985

**Published:** 2022-09-23

**Authors:** Alexander Woywodt, Raman Dhanda, David van Dellen, Beng So, Rowland J. Bright-Thomas

**Affiliations:** ^1^Department of Renal Medicine, Lancashire Teaching Hospitals NHS Foundation Trust, Royal Preston Hospital, Preston, United Kingdom; ^2^Manchester Centre for Transplantation, Manchester University NHS Foundation Trust, Manchester, United Kingdom; ^3^Manchester Adult Cystic Fibrosis Centre, Manchester University NHS Foundation Trust, Manchester, United Kingdom

**Keywords:** cystic fibrosis, kidney transplantation, pancreas transplantation, diabetes, living donor transplantation, chronic kidney disease

## Abstract

Cystic fibrosis (CF) is a multisystem disorder and represents the most common inherited condition leading to death in Western countries. Previous reports of chronic kidney disease (CKD) in CF focus on cases post lung, or other solid organ, transplantation but CKD in CF patients pre transplantation is increasingly recognized as a challenging complication of CF. CKD can evolve as a sequel to acute kidney injury for example after prolonged treatment with aminoglycoside antibiotics during episodes of infection. Nephrolithiasis, diabetic nephropathy and a variety of glomerular lesions, such as amyloidosis and Immunoglobulin A nephropathy are also seen. Muscle depletion is common in CF, hence creatinine-based estimates of kidney function may underestimate the degree of renal impairment and lead to delayed diagnosis and management. Improved treatment options for CF patients have resulted in a sustained increase in life expectancy with increasing numbers of CF patients with CKD approaching end-stage renal failure prior to consideration of lung transplantation. We believe that kidney or combined kidney-pancreas transplantation are under-utilized in this population. We provide a brief primer on the landscape of CF and CKD and discuss transplant options. Suitable patients with CF and advanced CKD should be formally assessed for kidney or kidney-pancreas transplantation.

## Introduction

Cystic fibrosis, the most common life-limiting genetic disorder in Caucasian populations, is a multisystem disease most frequently associated with repeated respiratory infections and progressive respiratory failure. The clinical course and prognosis of CF have changed considerably with modern treatment and median life expectancy is now >50 years. This is likely to increase further with the recent advent of highly effective CFTR modulator therapy (1, 2). Acute kidney injury is well-described in Cystic Fibrosis (CF) but chronic kidney disease (CKD) has only recently received detailed attention. Mechanisms of CKD in CF are multifactorial and include nephrolithiasis, aminoglycoside toxicity (3), diabetic kidney disease, IgA nephropathy, and amyloidosis (4). A 2018 study in 181 CF patients in Denmark showed a CKD prevalence of 2.7% which increased to 11% after inclusion of lung transplant recipients (5). Recent data from UK CF Registry shows an incidence of kidney stones of 2.2% and renal failure of 1.4% (6). Increasing numbers of such patients will therefore reach advanced stages of CKD and require complex discussions around renal replacement therapy and kidney transplantation. Like many other centers we have not routinely considered CF patients as candidates for kidney or combined kidney and pancreas transplantation. Here, we report our experience with a small number of successful transplants, describe evolving clinician perceptions and listing practice in our region and propose a more proactive approach to evaluating and waitlisting these uniquely complex patients.

## Case vignette

A 39-year-old man with CF presents with progressive chronic kidney disease in Summer, 2021. His serum creatinine is 322 μmol/l, with an estimated glomerular filtration rate (GFR) of 20 ml/min and a Cystatin C GFR of 13 ml/min. There is significant proteinuria of 1.7 g/l. He is under regular care by a nephrologist and underwent two kidney biopsies which showed IgA nephropathy. Contributing factors include cumulative exposure to repeated courses of intravenous and nebulised aminoglycoside antibiotics. There is also a remote history of kidney stones but repeat ultrasound showed normal sized kidneys with some small cysts and no stones. He also has insulin-dependent CF related diabetes (CFRD) with a HbA1c of 50 mmol/mol. His functional status is excellent, and he works full time as a plant operator for a construction company. He has had no CF-related hospital admissions for over 2 years. He has multi-lobar bronchiectasis and is chronically colonized by *Pseudomonas aeruginosa*, but pulmonary function is stable with a forced expiratory volume of 2.2 l (60% predicted) and vital capacity of 4.25 l. He is 179 cm tall and weighs 90 kg, resulting in a body mass index of 28 kg/m^2^. He does have cystic-fibrosis related liver disease (CFRD) but no cirrhosis or portal hypertension and is regularly assessed by a hepatologist His medication includes Insulin, daily Azithromycin, Colecalciferol, Dapagliflozin, Fexofenadine, sodium bicarbonate, theophylline, Tranexamic acid, Irbesartan, Ursodeoxycholic acid, Omeprazole, Salbutamol and Seretide inhalers. He is prescribed alternating months nebulised antibiotics Meropenem and Aztreonam and long term nebulised mucolytic Dornase Alfa. He has been prescribed the cystic fibrosis transmembrane conductance regulator (CFTR) modulator therapy Kaftrio (tezacaftor/ivacaftor with elexacaftor) for 12 months with 9% increase in lung function. What transplant strategy is appropriate to address this man's progressive chronic kidney disease?

## Chronic kidney disease in CF

The clinical scenario described above is becoming more common in our clinical practice and CKD is now a well-described complication of patients with CF (7). Mechanisms include tubulointerstitial disease caused by nephrolithiasis, nephrotoxicity due to use of aminoglycoside antibiotics and a variety of glomerular lesions such as Immunoglobulin A nephropathy or amyloidosis (7). Another important mechanism is diabetic nephropathy given that CF-related diabetes mellitus (CFRD) is now present in 40% of adults (8). The prevalence of CFRD is expected to increase with increased longevity of CF patients overall. The continually increasing patient survival is one of the main reasons why CKD care is gaining more relevance in CF (9). Median predicted life expectancy in CF patients is currently over 50 years in both UK and US CF populations (6) and is expected to increase even further with the increasing use of CFTR modulator therapy which is now licensed for over 90% of patients with CF in the UK and the US.

Close monitoring of kidney function is recommended for CF patients (10) but assessment of CKD in patients with CF can be challenging. Creatinine-based estimation of kidney function regularly under-estimates the severity of renal failure in CF patients due to low muscle mass. This effect may contribute to late referral to nephrologists and therefore to delayed decision making and preparations for renal replacement therapy. Cystatin C-based estimation of kidney function has been advocated by some authors (11) although this suggestion has been disputed (12). The assessment of CF patients with CKD is further hampered by the fact that these patients undergo renal biopsy less often than other patients with CKD with consequent less information available regarding etiology, possible treatment and prognosis (4).

Discussions about renal replacement therapy in CF patients are often difficult not only due to the medical complexity of these cases but also because the outcome of such patients on dialysis has received very little attention in the literature ever since a first case surviving maintenance dialysis long term was described in 1989 (13). There is also very little in the literature on CF patients with advanced kidney failure who decide for conservative care i.e., who make an informed decision against dialysis. Based on our own experience we believe that the paucity of data on renal replacement therapy in CF beyond the scenario of acute kidney injury (14) is caused by the fact that many patients with CF on maintenance dialysis don't do well. Peritoneal dialysis can further compromise the respiratory situation when the abdomen is filled with fluid whereas haemodialysis is often complicated by access issues typically following multiple lines, port devices and peripheral cannulas patients have had by the time patients reach advanced stages of CKD. Finally, it is important to appreciate that adding dialysis to the very high treatment burden in CF also has significant effects on quality of life, wellbeing, and treatment compliance.

## CKD following lung transplantation in CF

CKD often worsens after lung transplantation in CF patients (5) and multiple factors can contribute (15). These include calcineurin inhibitor toxicity as well as tubulointerstitial disease and accelerated vascular disease (16). Polyoma virus infection of the native kidneys after lung transplant has also been described (17). CF patients have the best prognosis of any group post lung transplant and up to 50% can expect to survive for more than 7 years (7). However, compared to other patient groups they appear to have an accelerated decline of kidney function (18). Female gender and increasing age have been described as risk factors (7). CF patients should therefore undergo detailed assessment of their renal status when lung transplantation is being considered (19). Older CF registry data report a 5-year risk of 58% of reaching CKD Stage G3 or worse with 10% of patients eventually requiring renal replacement therapy (20). The situation seems to have improved somewhat since the switch in standard immunosuppression from Cyclosporine to Tacrolimus (18). The historical outcome of CF patients post lung transplant on dialysis is generally poor with older reports describing a Median survival of 5 months (21). Technical challenges are similar to other CF patients whereby peritoneal dialysis is not usually possible as it would lead to respiratory compromise when the abdomen is filled whereas vascular access for hemodialysis is often hampered by multiple previous attempts to access peripheral and central veins (22). Moreover, on haemodialysis these patients don't tend to tolerate fluid overload during the inter-dialytic interval.

## Primary kidney transplantation in CF

CF patients have long been considered to have a very high risk with kidney transplantation with concerns around chronic respiratory infections, significant co-morbidity and performance status overall. This widely held belief has been challenged (23) and our own perceptions started to evolve starting with successful kidney transplant outcome in a 45-year-old man with CF described elsewhere (24). In total we have now transplanted two CF patients and waitlisted two more which documents how our listing practice has evolved. The 45-year-old male described previously (24) had a successful renal transplant in 2015 followed by successful pancreas transplant in 2019 and remains well at the time of writing. We next listed a 27-year-old male with CF who was on in-center haemodialysis due to IgA nephropathy (Table 1). While younger than the first case he had recurrent haemoptysis, pulmonary oedema with severely reduced LV ejection fraction on echo and an infected knee joint which resulted in a period of suspension from the waiting list in 2019. After careful deliberation we decided to reactivate him on the wait list and he was transplanted in January 2020 with good outcome, no evidence of sepsis, resolution of pulmonary oedema and subsequent normalizing of cardiac ejection fraction. We then listed a 39-year-old female CF patient who at the time of writing remains active on the waiting list but has more severe lung disease than the other patients we had listed before.

**Table 1:** CF patients listed and/or transplanted in our centre.

**Patient**	**Age/ gender**	**Cause of kidney disease**	**Co-morbidity**	**Respiratory situation at time of transplant or listing**	**Outcome**
#1	45/M	Solitary kidney, presumed hypertensive and diabetic nephropathy (no biopsy). On in centre haemodialysis	Pancreatic insufficiency, diabetes	Multilobar bronchiectasis with chronic Pseudomonas infection; FEV1 41% predicted, pleural effusions. Recurrent fluid overload	Kidney transplant 2015. Excellent kidney function, serum creatinine 75 umol/l. Subsequent pancreas-after-kidney transplant with good outcome in 2019. Well as of summer 2022
#2	27/M	Biopsy proven IgA nephropathy. On in centre haemodialysis	History of bowel obstruction. pancreatic insufficiency Dilated cardiomyopathy with previously severe LV impairment, currently mild LV impairment, EF 49%	Recurrent haemoptysis and chronic colonisation with Pseudomonas. Normal FEV1	Listed 10/2018 and transplanted from a deceased heart beating donor 1/2020. Good transplant function, serum creatinine 174 umol/l as of February 2022. No hospital admissions since kidney transplant
#3	29/F	Chronic kidney disease CKD stage G5 (cystatin GFR 14 ml/min) due to diabetic nephropathy and nephrolithiasis. On in centre haemodialysis	CF related diabetes and pancreatic insufficiency	Chronic pseudomonas infection; Bronchial artery embolization 2002 for haemoptysis. FEV1 0.95 l (43% predicted)	First listed for kidney transplantation in 5/2021. Active on the wait list as of early summer 2022
#4	41/M	Chronic kidney disease CKD stage G5 (Cystatin C GFR 13 ml/min), due to nephrolithiasis and use of aminoglycoside antibiotics (no biopsy). Not yet on dialysis	CF related diabetes. mild splenomegaly and cystic-fibrosis related liver disease without evidence of cirrhosis or portal hypertension	Stable - FEV1 2.5l	First listed for combined kidney and pancreas transplantation in February 2022. Active as of August 2022

Transplant physicians often consider CF patients to be at high risk of severe respiratory infections after kidney transplantation but these patients are immunocompetent in principle and have not been shown to be at additional risk from immunosuppression in the context of lung or liver transplantation. Of note, we (24) and others (23) have described improved lung function as a result of kidney transplantation and also a reduction in the need for antibiotics post renal transplant. With growing regional experience of CF and kidney transplantation overall we now regularly consider patients with CF for a kidney transplant. We have also taken steps to alert nephrologists and CF physician colleagues to the fact that we no longer consider CF as a contra-indication to transplantation, and we have asked for colleagues to ensure transplantation has been considered and discussed in suitable CF patients. We emphasize the importance of individual assessment, considering also the predicted overall survival of a patient.

It is also worth considering the timing of transplant listing in relation to the degree of renal failure. We would argue that these patients should be assessed for kidney transplant options earlier than other patients with CKD because they are almost uniquely complex among patients referred for kidney transplantation. We emphasize the importance of detailed assessment and multi-disciplinary approach all of which takes time. It is also important to try as much as possible to optimize not only the respiratory situation but also other co-morbidities prior to transplantation, including the nutritional status. Patients not thought suitable for transplantation will also require detailed input from the multi-disciplinary team in terms of discussing dialysis options and palliative care (25), if appropriate.

It is also clear that psychologically the prospect of a kidney transplant will be daunting for many CF patients who on average suffer from a high burden of healthcare interventions, interactions and medications (26) While these patients have significant co-morbidities, they are in general younger than many other most renal transplant recipients and candidates and often highly motivated. In addition, CF patients often have a well-developed relationship with their CF care providers and support structures are already in place. This is likely one of the reasons why CF patients have the best survival of any patient subgroup following lung transplantation with a median survival of 8.9 years (27).

Time on the waiting list for CF patients for kidney transplantation in relation to the severity of their respiratory situation also deserves consideration. Three of the four patients we have listed had stable respiratory status and preserved pulmonary function. Patient 3, however, had more severe lung disease but did not require a lung transplant yet. We emphasize the importance of regular and close dialogue with respiratory physicians and shared decision making. It is important to not delay lung transplantation to suitable CF patients and multidisciplinary dialogue should agree priorities in this regard. Combined kidney and lung transplantation has been described in a small number of patients (28, 29) but it is currently difficult to routinely recommend this approach given the magnitude of the surgical intervention and paucity of published data.

The anesthetic management of patients with CF undergoing kidney transplantation also deserves consideration (30). In the cases described here the anesthetic team's main concern was the effect of a general anesthetic and intubation on patients respiratory status and we considered spinal (31) and also epidural (32) anesthesia. We are currently evaluating these options but cannot report any experience with this approach. We emphasize the importance of multidisciplinary peri-operative management (33) and detailed pre-operative anesthetic assessment involving the patient's CF team who usually know their patients extremely well. The risk of respiratory complications and also that of postoperative distal intestinal obstruction syndrome (DIOS) should be discussed and become part of the consent process. Every effort should be made to optimize the respiratory situation prior to transplant.

One shortcoming of our experience is the lack of live donor transplants. It is reasonable to suggest that a live donor kidney transplant will have significant advantage in this unique population as it will enable all teams to provide multidisciplinary support in an elective and planned setting. We therefore believe that assessment for kidney transplantation in CF should occur early on for example when their glomerular filtration rate (preferably estimated independent of muscle mass) has fallen below 25 ml/min, not least to give time for discussions around live donation. These discussions are likely complex and should also bear in mind the very substantial care burden, fear and uncertainty for families at this point in time. Finally, we have not so far transplanted or waitlisted any CF patients with CKD following lung transplantation, but we note that others have described a survival benefit in selected patients (34).

## CF and combined kidney and pancreas transplantation

Given the widely held concerns around kidney transplantation it is not surprising that combined kidney pancreas transplantation (SPKT) in CF patients with renal failure and diabetes is even less often considered let alone carried out with only four cases of SPKT reported in US CF population from 1987 to 2014 (35). Most of these transplants occurred following previous lung transplantation. The issue is highly relevant given that diabetes is present in up to 50% of adult patients with CF in contemporary studies (36). Cystic fibrosis-related diabetes (CFRD) is different in terms of pathophysiology (37), phenotype and treatment from other forms of diabetes and its presence is associated with worse prognosis (38). The prevalence of CFRD is likely to increase due to the increased life expectancy of CF patients. Fridell et al. described successful SPKT after lung transplant in CF (39) and emphasize the beneficial effects on exocrine pancreas insufficiency (40). This may be potentiated by the technical consideration of a more proximal enteric anastomosis with aim of maximizing the potential benefit of exocrine function.

The 39-year-old man with CF and CF-related insulin-dependent diabetes described in our case vignette was listed for combined kidney and pancreas transplantation after risks and benefits were considered and explained to him. A conscious decision against combined kidney islet cell transplantation was made in view of his CF-related liver disease. He was listed for a combined kidney and pancreas transplant in February 2022 and remains active on the waitlist as of August, 2022. We could not find any cases of combined kidney and islet cell transplantation (SIK) in a CF patient in the literature. This could be explained by the fact that SIK in itself is still a relatively novel technique or by the fact that many CF patients have CF related liver disease thus increasing the risk of SIK. It is also the case that many CF patients have concurrent exocrine pancreatic insufficiency which may lead transplant teams to choose SPK over SIK in these patients.

Few simultaneous lung and pancreas transplants have been performed and sequential transplantation of the lung first and pancreas later on may allow the recipient to recover in between the two major interventions (40). Combined liver and pancreas transplantation may be an option for patients with CF, diabetes and CF-related liver disease (41). Bandesma et al. reported a multi-center survey that featured 8 such patients (42). Very recently, combined lung, kidney and liver transplantation from a single deceased donor has been described in a 23 year old CF patient with renal failure due to IgA nephropathy and concurrent CF-related liver disease (43). A staged approach with liver and lung transplantation followed by kidney transplant later has also been reported (44). It is worth noting that the transplant community seems to be increasingly willing to consider multi-organ transplantation in CF. We speculate that with advances in donor optimization, surgical technique and peri-operative anesthetic management, confidence in offering multi organ transplants to these high-risk patients may increase further.

**Figure 1:**
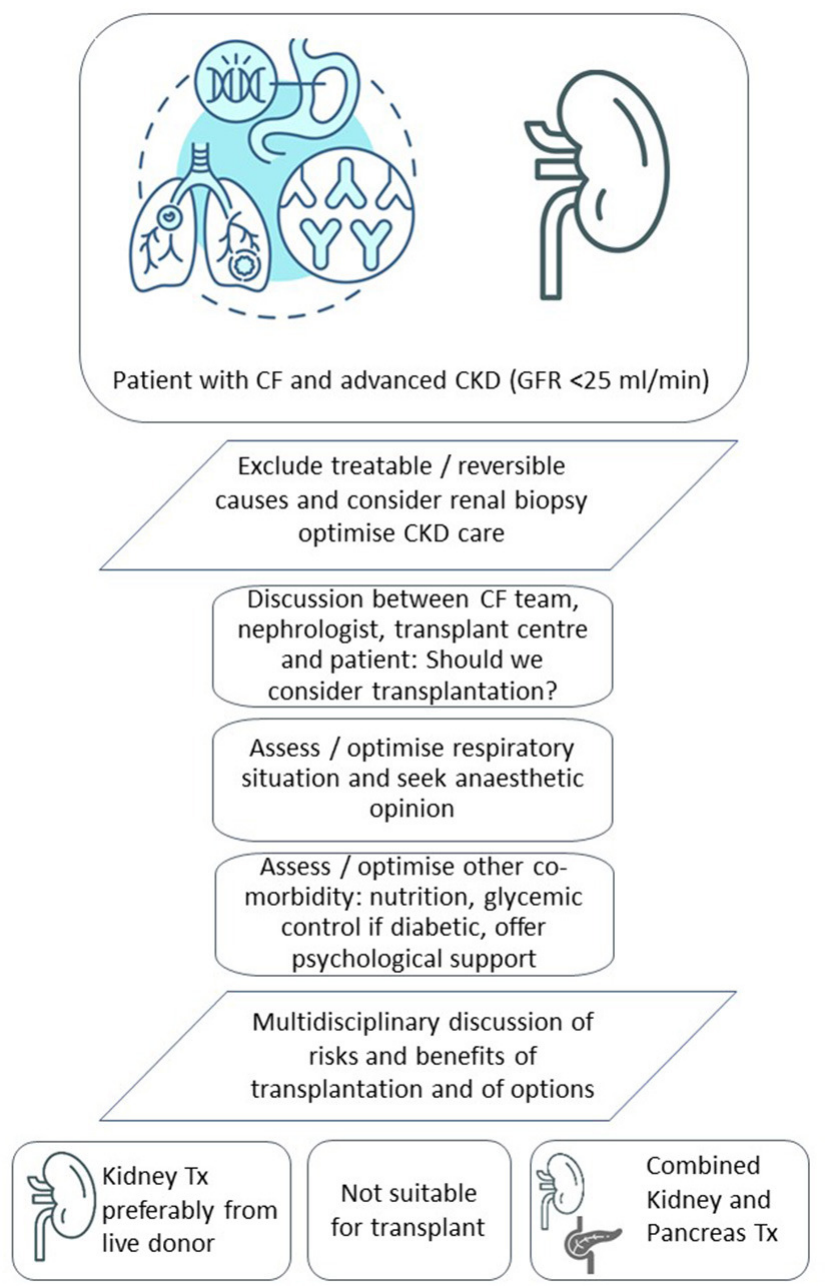
Transplant options and suggested decision making in patients with CF and advanced CKD.

In conclusion, CF patients with advanced CKD but good functional performance and without other contraindications should be assessed for kidney transplantation. Live donation should be considered wherever possible and the advantages of live donation in patients with CF should be explained to patients and families. Younger patients with concomitant CFRD should be considered for combined kidney pancreas transplantation, if appropriate. Both kidney and combined kidney and pancreas transplantation may be under-utilized in CF perhaps due to a degree of risk-averse behavior in the transplant community overall (45). Failure to appreciate the poor outlook of these patients on dialysis and the impact on quality of life through the additional burden of care associated with dialysis itself may also play a role. We do not advocate that all or most patients with CF and advanced CKD should be listed for a kidney or kidney and pancreas transplant. Instead, we emphasize the importance of early individual patient assessment, multidisciplinary decision making and dialogue with the patient. Figure 1 shows our current care pathway for CF patients with advanced CKD. Respiratory physicians and nephrologists caring for CF patients should consider more structured approaches and seek dialogue to ensure transplantation has been considered in suitable patients. This is particularly relevant where CF physicians, nephrologists and transplant teams are not co-located on the same site which is often the case in CF when respiratory specialist care is delivered in a small number of regional units. Further research should include a dedicated registry of kidney and kidney pancreas transplantation in CF, as well as detailed outcome data of CF patients on dialysis. Such data would help us to better identify CF patients with advanced kidney failure who will benefit from kidney and kidney-pancreas transplantation.

## Data availability statement

The original contributions presented in the study are included in the article/supplementary material, further inquiries can be directed to the corresponding author.

## Ethics statement

Ethical review and approval was not required for the study on human participants in accordance with the local legislation and institutional requirements. Written informed consent for participation was not required for this study in accordance with the national legislation and the institutional requirements. Written informed consent was obtained from the individual(s) for the publication of any potentially identifiable images or data included in this article.

## Author contributions

AW was involved in the medical transplant assessment of the patient in the case vignette and wrote the first draft of the manuscript with RB-T. DD was involved in the surgical transplant assessment of the patient and contributed to the manuscript. RD has performed pancreas after kidney transplant in one CF patient, assessed another patient for potential SPKT and contributed to the manuscript. BS looked after the patients in clinic and contributed to the manuscript. RB-T was involved in the care of all the CF patients at the Manchester Adult Cystic Fibrosis Center. All authors have seen and approved the final version of the manuscript.

## Conflict of interest

The authors declare that the research was conducted in the absence of any commercial or financial relationships that could be construed as a potential conflict of interest.

## Publisher's note

All claims expressed in this article are solely those of the authors and do not necessarily represent those of their affiliated organizations, or those of the publisher, the editors and the reviewers. Any product that may be evaluated in this article, or claim that may be made by its manufacturer, is not guaranteed or endorsed by the publisher.
